# Precise Measurement of Long-Range Heteronuclear Coupling Constants by a Novel Broadband Proton–Proton-Decoupled CPMG-HSQMBC Method

**DOI:** 10.1002/chem.201405535

**Published:** 2015-01-08

**Authors:** István Timári, Tünde Z Illyés, Ralph W Adams, Mathias Nilsson, László Szilágyi, Gareth A Morris, Katalin E Kövér

**Affiliations:** [a] Department of Inorganic and Analytical Chemistry, University of DebrecenEgyetem tér 1, 4032 Debrecen (Hungary) E-mail: kover@science.unideb.hu; [b] Department of Organic Chemistry, University of DebrecenEgyetem tér 1, 4032 Debrecen (Hungary); [c] School of Chemistry, University of ManchesterOxford Road, Manchester, M13 9PL (UK)

**Keywords:** HSQMBC, heteronuclear coupling constants, NMR spectroscopy, proton–proton decoupling, structure elucidation

## Abstract

A broadband proton–proton-decoupled CPMG-HSQMBC method for the precise and direct measurement of long-range heteronuclear coupling constants is presented. The Zangger–Sterk-based homodecoupling scheme reported herein efficiently removes unwanted proton–proton splittings from the heteronuclear multiplets, so that the desired heteronuclear couplings can be determined simply by measuring frequency differences between singlet maxima in the resulting spectra. The proposed pseudo-1D/2D pulse sequences were tested on nucleotides, a metal complex incorporating P heterocycles, and diglycosyl (di)selenides, as well as on other carbohydrate derivatives, for the extraction of ^*n*^*J*(^1^H,^31^P), ^*n*^*J*(^1^H,^77^Se), and ^*n*^*J*(^1^H,^13^C) values, respectively.

## Introduction

Long-range heteronuclear coupling constants, ^*n*^*J*(^1^H,X), are invaluable tools for stereochemical and conformational analysis of synthetic organic molecules[1] and natural products,[2] and complement the information gained from proton–proton coupling constants and NOE data.[3] Even though many different approaches to their measurement have been proposed over the last two decades,[4, 5] measurement of ^*n*^*J*(^1^H,X) values is still not straightforward and is therefore relatively unexploited in structural studies on molecules.

Among the methods reported in the literature, HETLOC[6, 7] and HSQC-TOCSY[8–11] experiments are particularly useful for the measurement of heteronuclear multiple-bond couplings of protonated heteronuclei, but they fail for nonprotonated (e.g., quaternary C) centers, or when proton–proton TOCSY transfer is not efficient. In contrast, HMBC (heteronuclear multiple-bond correlation)[12–14] and HSQMBC (heteronuclear single quantum multiple-bond correlation)[15] methods and their variants are applicable regardless of the protonation state of the heteronucleus. However, a common drawback of HMBC- and HSQMBC-type approaches is that during the long (ca. 70–90 ms) coupling evolution period, the homonuclear proton–proton and proton–heteronucleus long-range coupling interactions evolve together, and thus mixed-phase signals arise in the resultant spectra. Therefore, extraction of the desired heteronuclear coupling constants often requires the use of complex fitting procedures; at worst, extraction of the coupling constants of interest may even be prevented if multiplets are severely distorted. To circumvent this limitation of the HSQMBC method, several modifications have been introduced into the long-range coupling-matched INEPT (insensitive nuclei enhanced by polarization transfer) component of the sequence, such as application of CPMG pulse trains,[16–18] selective and band-selective 180° proton pulses,[19, 20] and a perfect echo element.[21] However, even in these amended variants, the resulting HSQMBC peaks appear with complex multiplet patterns in which the undesired proton–proton splittings are superimposed on the antiphase doublets originating from the active heteronuclear coupling interactions. Thus, the evolution of proton–proton couplings during acquisition, which results in complex antiphase multiplets, can impede the extraction of the heteronuclear coupling constants.

Recently, it has been shown that proton–proton splittings can be eliminated from HSQMBC spectra by applying band-selective homonuclear decoupling to spectral regions with nonmutually coupled proton sites.[22] However, application of this scheme is limited to molecules with specific types of structure, for example, peptides.[22] Homonuclear broadband-decoupled ^1^H experiments have also been used for the measurement of heteronuclear coupling constants of compounds containing highly abundant heteronuclei.[23, 24]

Herein, we report a novel broadband proton–proton-decoupled CPMG-HSQMBC method for the simple and precise measurement of long-range heteronuclear coupling constants. In the proposed experiment, the undesired proton–proton splittings are eliminated with the aid of a broadband homodecoupling scheme based on the Zangger–Sterk (ZS) principle;[25] as a result, only evolution of heteronuclear couplings is active during acquisition. The desired multiple-bond heteronuclear couplings can thus be extracted simply by measuring the frequency differences between the peaks of pure antiphase doublets. In addition, the relative signs of coupling constants can be determined from the characteristic sign pattern (up/down or down/up) of the antiphase signals.

## Results and Discussion

Pure shift (PS) methods that suppress the effects of proton–proton scalar couplings in the directly detected proton dimension provide simplified spectra and increased resolution, and have attracted considerable attention in recent years.[26–44] Common broadband proton–proton decoupling methods include those based on the bilinear rotation decoupling (BIRD)[32–37] and ZS pulse-sequence modules.[26–31] The former utilizes an isotope-selection approach: depending on the relative phases of the individual proton pulses of BIRD modules,[45] protons that are either directly attached or not attached to isotopically dilute spins (e.g., ^13^C, ^15^N) can be selectively and independently inverted. The ZS method[25] uses spatially and frequency-selective excitation by combining a selective 180° proton pulse with a weak magnetic field gradient.

**Figure 1:**
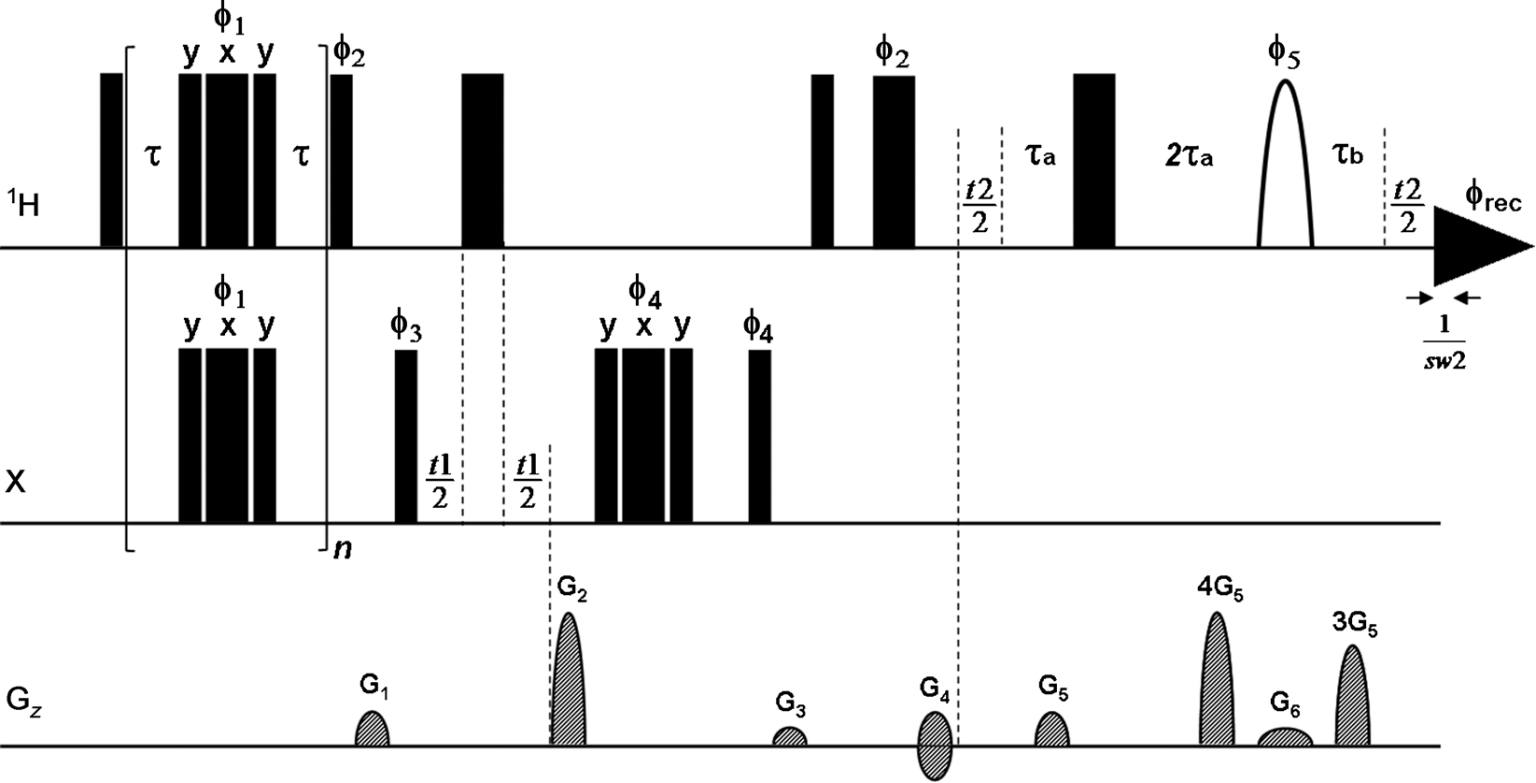
Pulse sequence scheme of the broadband proton–proton-decoupled CPMG-HSQMBC experiment designed for the measurement of long-range heteronuclear coupling constants. Narrow and wide filled bars correspond to 90 and 180° pulses, respectively, with phase *x* unless indicated otherwise. The selective shaped proton pulse is shown as a half-ellipse. *φ*_1_ is incremented according to XY-16 cycles within the CPMG sequence; thus, *n* should ideally be adjusted to a multiple of 16. Other phases are *φ*_2_=*y*; *φ*_3_=*x*, −*x*; *φ*_4_=*x*, *x*, −*x*, −*x*; *φ*_5_=*x*, *x*, *x*, *x*, *y*, *y*, *y*, *y*; and *φ*_rec_=*x*, −*x*, −*x*, *x*, −*x*, *x*, *x*, −*x*. Delays are set as follows: *τ*=120–150 μs, *τ*_a_=1/(4*sw2), *τ*_b_=1/(4*sw2)−4/sw. Coherence order selection and echo–antiecho phase-sensitive quadrature detection in the X dimension are achieved with gradient pulses G_2_ and G_4_ in the ratio 80:20.1 for ^13^C, 80:32.3846 for ^31^P and 80:15.257 for ^77^Se, respectively. Purging gradient pulses G_1_ and G_3_ are set to 19 and 10 % of maximum gradient strength (53 G cm^−1^). Coherence selection gradient pulses used in the extra proton–proton-decoupled dimension have G_5_=18 %. Sine-bell-shaped gradient pulses of 1 ms duration are utilized, followed by a recovery delay of 200 μs. The slice-selection gradient (G_6_) is adjusted for each molecule as reported in the legends to the respective figures.

Herein, we propose a broadband proton–proton-decoupled CPMG-HSQMBC experiment that utilizes an improved version of the ZS broadband homodecoupling scheme. In the pulse sequence (Figure  1), broadband proton decoupling in the directly detected proton dimension is achieved by replacing the conventional free induction decay (FID) acquisition of the CPMG-HSQMBC sequence with a second evolution time *t*_2_, during which a hard 180° proton pulse and a weak gradient field under a selective 180° proton pulse are applied in succession, followed by acquisition of a chunk of FID *s*(*t*_3_). The combination of a weak gradient field with a selective 180° proton pulse is used to restrict the measurement of the signal from each different chemical shift in the spectrum to a different horizontal slice through the sample. The combination of selective and nonselective 180**°** proton pulses then ensures that all protons that are off-resonance are inverted, while the on-resonance protons (and the undisturbed heteronuclei) remain unaffected. Consequently, the net effect is to allow the continuous evolution of the proton chemical shift and the heteronuclear coupling throughout *t*_2_ and to refocus the evolution of the undesired proton–proton couplings at the midpoint of the acquisition of a FID chunk. Because proton–proton couplings evolve much more slowly than chemical shifts, FID chunks *s*(*t*_3_) can be typically acquired with a duration of 10–25 ms, matched to the increment 1/sw2 used for the second evolution time *t*_2_. During processing, prior to regular 2D FT a pseudo-2D dataset (interferogram) is constructed by concatenating all the data chunks recorded to give a synthetic FID without homonuclear *J* modulation.

However, all ZS-type experiments, including our new method, involve a trade-off between the sensitivity, the minimum frequency difference to be decoupled, and the range of chemical shifts to be covered. Typically, the sensitivity of these proton-decoupled experiments is about 1–10 % of that of the conventional analogue.[46] The actual sensitivity loss depends on the choice of experimental parameters for slice selection, which in turn depend on the nature of the spin systems involved. For efficient homonuclear decoupling, the selective pulse should be selective enough to affect only one coupling partner. However a soft pulse with narrow bandwidth generates a signal from only a thin slice of the sample, and hence the sensitivity of the experiment is reduced. The range of chemical shifts to be decoupled determines the strength of the gradient required, so increasing the shift range again reduces the slice thickness and hence the sensitivity. Thus, the sensitivity of a ZS experiment is directly proportional to the bandwidth of the selective pulse, and inversely proportional to the strength of the slice-selection gradient. In practice, these two parameters should be carefully chosen for a given sample, for example, with the help of the much quicker 1D PS (ZS- decoupled) ^1^H experiment.[27]

**Figure 2:**
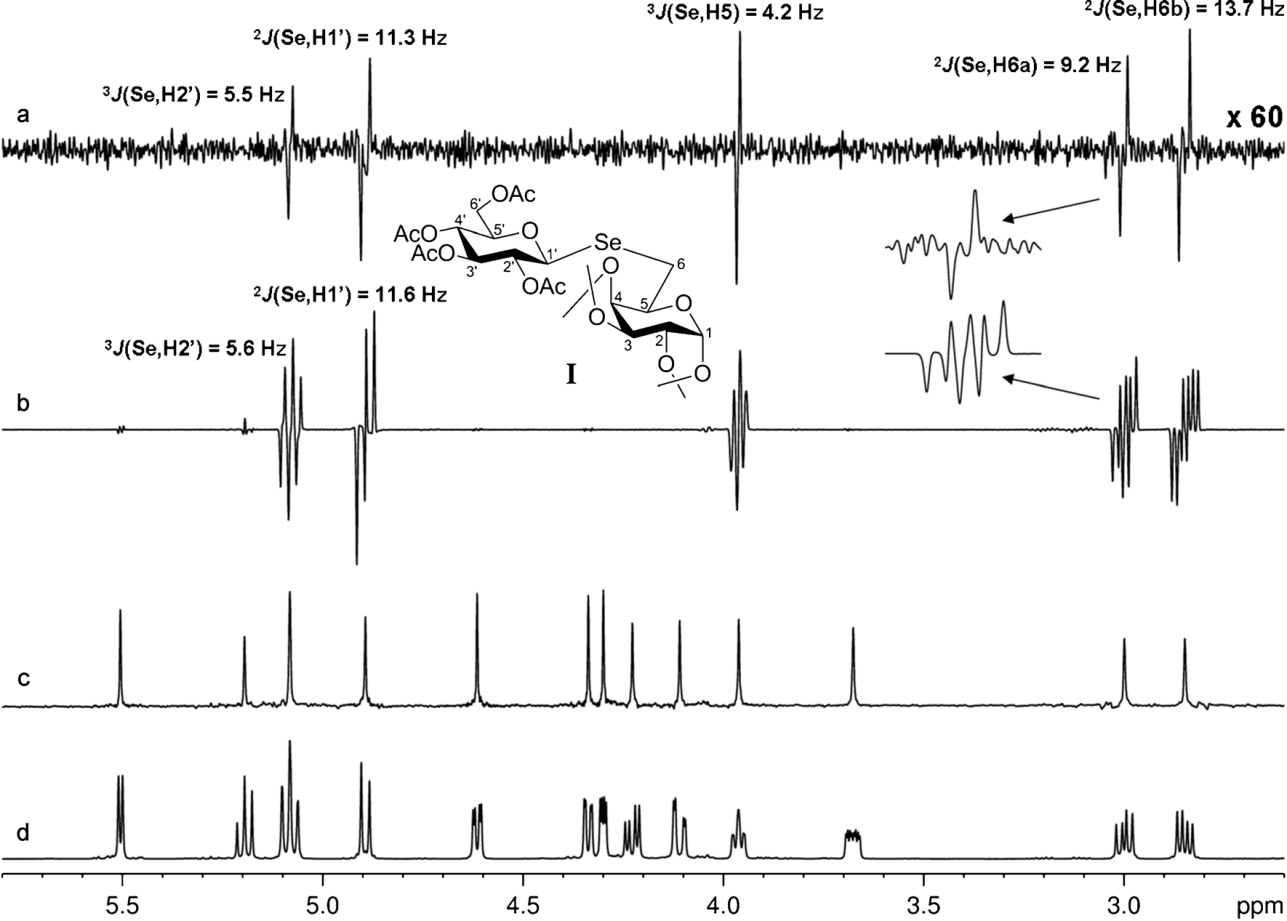
Comparison of CPMG-HSQMBC spectra obtained for diglycosyl-selenide I, with (a) and without (b) broadband proton–proton decoupling. Measurement times were 5.9 h (a) and 45 min (b). The homonuclear decoupled pseudo-1D spectrum (a) was collected by using the sequence of Figure  1 with the incremented delay *t*_1_ replaced by a constant delay of 3 μs. Representative ZS-based PS ^1^H (c) and normal ^1^H NMR spectra (d) are also shown. All spectra in this figure were recorded with a spectral width of 6.0371 ppm. In the broadband proton–proton-decoupled spectra (a, c), an RSNOB selective 180° proton pulse[47] of duration 46.64 ms and bandwidth 50 Hz under a slice-selection gradient (G6) of 1 % of the maximum gradient strength was used. These spectra (a, c) were acquired with number of *t*_2_ increments (i.e., number of FID chunks)=32, duration of FID chunk=16.56 ms, number of complex data points of constructed FID in ^1^H dimension=3200, relaxation delay=2 s, number of scans=128 (a) and 4 (c). Spectrum b) was collected with number of complex data points=4096, relaxation delay=1.7 s, and number of scans=1024, by using the conventional CPMG-HSQMBC sequence.[17] The HSQMBC experiments (a, b) were recorded with 81.7 ms of heteronuclear coupling evolution during the initial CPMG-INEPT step.

To validate the performance of the new method, we first tried a pseudo-1D version of the proton-decoupled CPMG-HSQMBC sequence on simple model compounds containing only one highly sensitive (^31^P: 391 times more sensitive than ^13^C) or one moderately sensitive (^77^Se: 3.15 times more sensitive than ^13^C) heteronucleus and an extensive set of mutually coupled protons. For example, Figure  2 shows the standard ^1^H, PS ^1^H, standard CPMG-HSQMBC,[17] and broadband proton–proton-decoupled CPMG-HSQMBC spectra of model diglycosyl selenide **I**. In the traditional CPMG-HSQMBC multiplets (Figure  2 b), in several cases the many in-phase proton–proton splittings severely compromise extraction of the multiple-bond ^1^H–^77^Se coupling constants from the complex (in- and antiphase) multiplets. In contrast, the pure antiphase doublets of the broadband proton–proton-decoupled CPMG-HSQMBC spectrum (Figure  2 a) allow the measurement of all desired heteronuclear couplings with ease and high precision. For comparison, if reliable measurement was feasible from the original CPMG-HSQMBC experiment, values of coupling constants were extracted from both the proton-coupled and the new proton-decoupled HSQMBC multiplets. The coupling constants obtained by the two different methods agree within experimental error, and this confirms that the proton–proton decoupling sequence applied during acquisition has no undesired effect on the measured multiplet splittings. According to our previous studies on other selenoglycosides, such coupling data are highly valuable and present a promising tool for the assessment of the glycosidic conformation around the C(1)–Se bond[48] and for the unambiguous stereospecific assignment of diastereotopic CH_2_ protons next to Se.[49]

**Figure 3:**
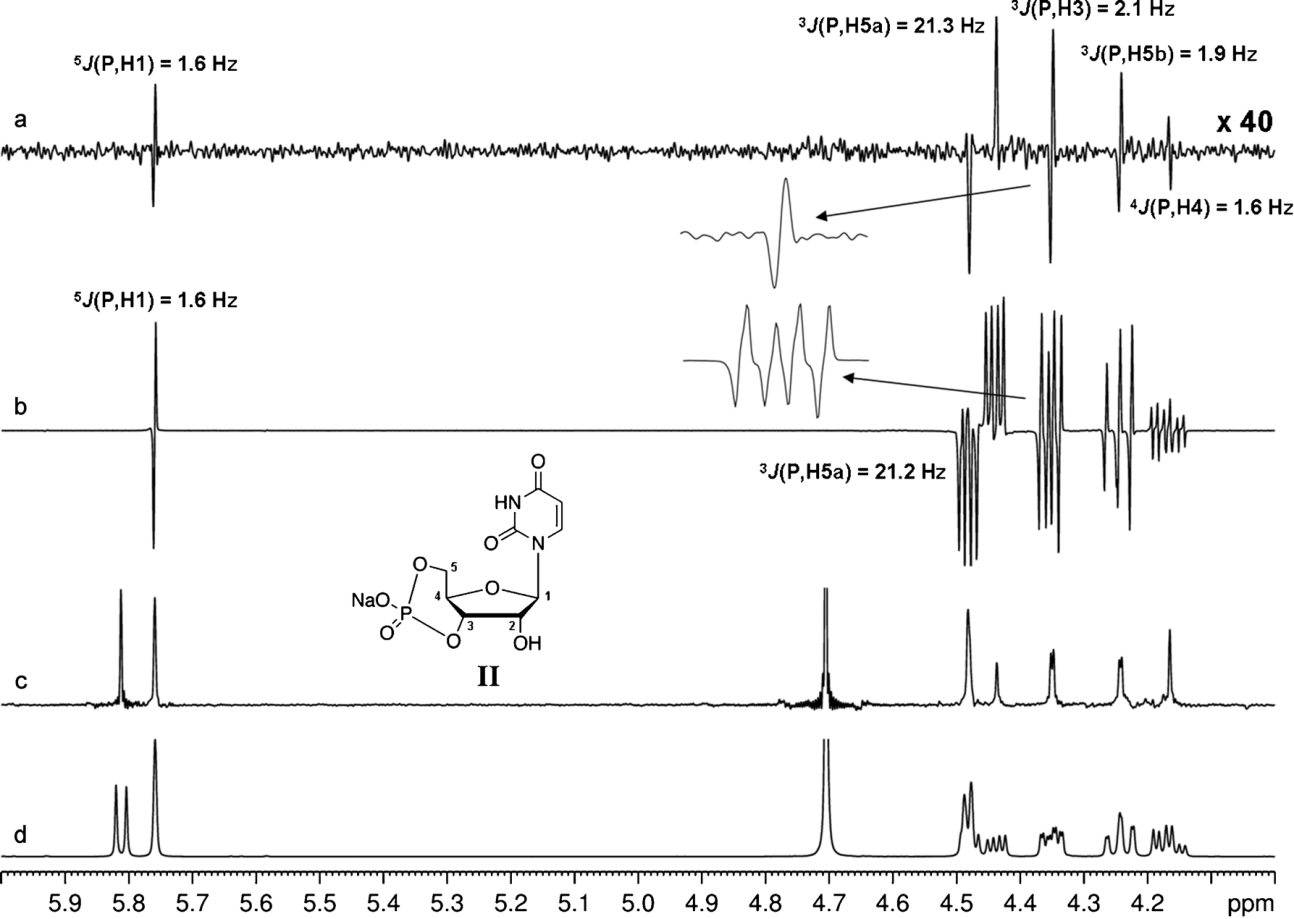
Comparison of CPMG-HSQMBC spectra obtained for II, with (a) and without (b) broadband proton–proton decoupling. Measurement times were 1.5 h (a) and 10 min (b). The homonuclear decoupled pseudo-1D spectrum (a) was collected by using the sequence of Figure  1 with the incremented delay *t*_1_ replaced by a constant delay of 3 μs. Representative ZS-based PS ^1^H (c) and normal ^1^H NMR spectra (d) are also shown. Spectra a), c), and d) were recorded with spectral widths=6.0371 ppm. In the cases of the broadband proton–proton-decoupled spectra (a, c), an RSNOB selective 180° proton pulse[47] of duration 93.28 ms and bandwidth 25 Hz under a slice-selection gradient (G6) of 1 % of the maximum gradient strength was used. These spectra (a, c) were acquired with number of *t*_2_ increments (i.e., number of FID chunks)=32, duration of FID chunk=16.56 ms, number of complex data points of constructed FID in ^1^H dimension=3200, relaxation delay=1.7 s, number of scans=32 (a) and 8 (c). Spectrum b) was collected with spectral width=9.9774 ppm, number of complex data points=16 384, relaxation delay=1.7 s, and number of scans=128 by using the conventional CPMG-HSQMBC sequence.[17] The HSQMBC experiments (a, b) were recorded with 81.7 ms of heteronuclear coupling evolution during the initial CPMG-INEPT step.

Test measurements were run to assess the scope of our method for the determination of other long-range heteronuclear coupling constants, such as ^*n*^*J*(^1^H,^31^P). For the biologically relevant nucleotide cUMP (**II**), all long-range ^1^H–^31^P coupling constants could be determined from the broadband proton–proton-decoupled CPMG-HSQMBC spectrum simply by measuring the frequency differences between the peaks of pure antiphase doublets (Figure  3 a), whereas the analysis of the conventional CPMG-HSQMBC multiplets (Figure  3 b) is not straightforward. Figure  3 a also illustrates that multiple-bond heteronuclear coupling constants ranging between 1.6 and 21.3 Hz can be measured in a single experiment with our new method. These results also clearly demonstrate that the proposed pulse sequence shown in Figure  1, together with the gradient-based coherence selection scheme, efficiently removes undesired coherences arising from any mismatch between the duration of the CPMG-INEPT delay and ^*n*^*J*(^1^H,X). It has been well demonstrated in the literature that ZS-based broadband proton–proton decoupling schemes can handle highly complex proton–proton-coupled spin networks, and this paves the way for the applicability and utility of our approach for studying more complex systems, as illustrated by the examples shown in Figures  2 and 3.

**Figure 4:**
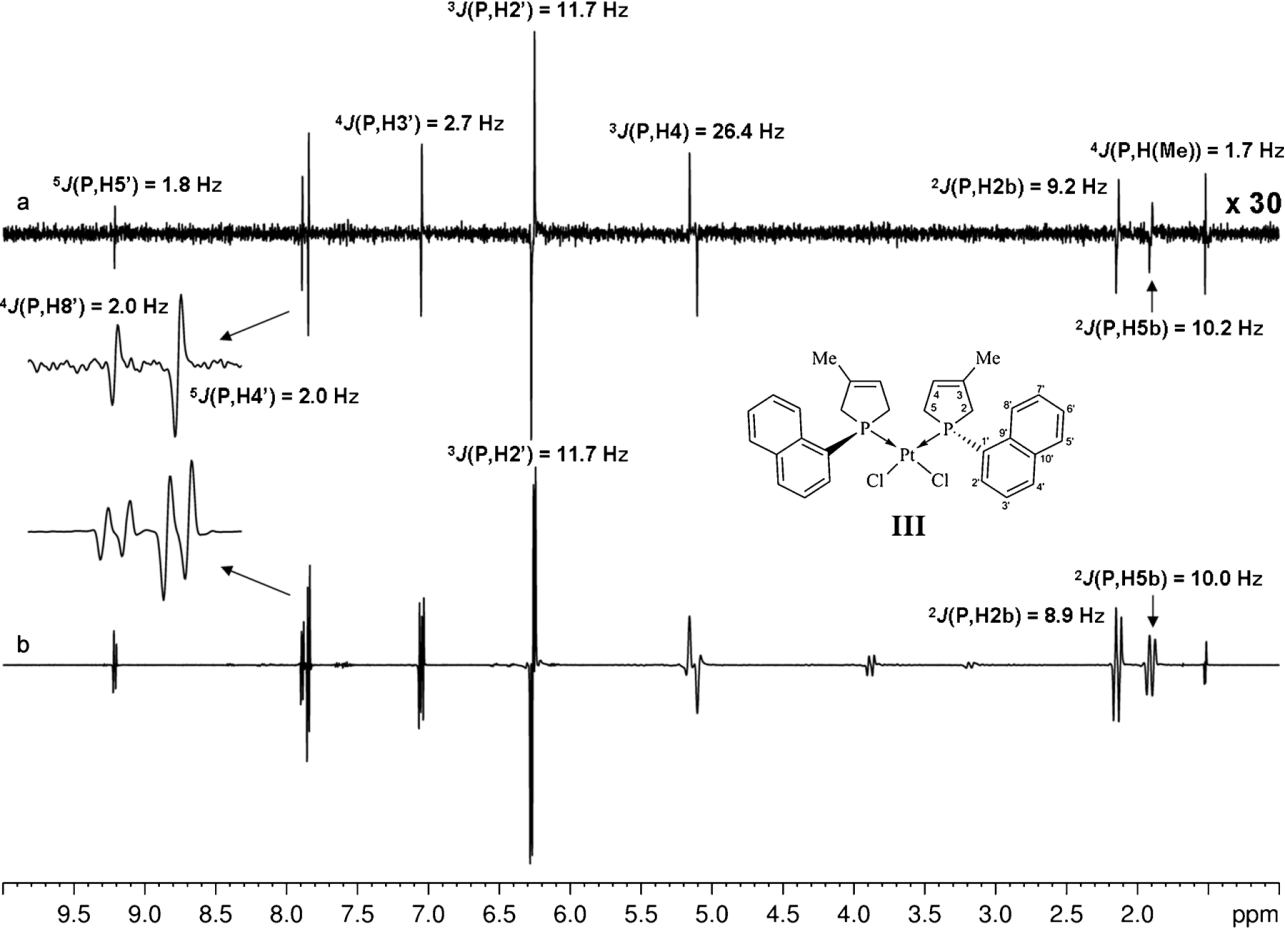
Comparison of CPMG-HSQMBC spectra obtained for III, with (a) and without (b) broadband proton–proton decoupling. Measurement times were 3.3 h (a) and 13 min (b). The homonuclear decoupled pseudo-1D spectrum (a) was collected by using the sequence of Figure  1 with the incremented delay *t*_1_ replaced by a constant delay of 3 μs, and using an RSNOB selective 180° proton pulse[47] of duration 23.32 ms and bandwidth 100 Hz under a slice-selection gradient (G6) of 1.6 % of the maximum gradient strength. This spectrum (a) was recorded with spectral width=9.9774 ppm, number of *t*_2_ increments (i.e., number of FID chunks)=32, duration of FID chunk=20.04 ms, number of complex data points of constructed FID in ^1^H dimension=6400, relaxation delay=1.7 s, and number of scans=96. Spectrum b) was acquired with spectral width=9.9774 ppm, number of complex data points=8192, relaxation delay=2 s, and number of scans=256 by using the conventional 1D CPMG-HSQMBC sequence.[17] The HSQMBC experiments (a, b) were recorded with 52.7 ms of heteronuclear coupling evolution during the initial CPMG-INEPT step.

Next, the usefulness of the proposed method was further illustrated with metal complex **III** incorporating P heterocycles (see Figure  4 for structure[50]). The broadband proton–proton-decoupled CPMG-HSQMBC spectrum clearly demonstrates that, if necessary, signals for an extensive range of chemical shifts can be recorded in a single experiment, and splittings measured by suitably adjusting the strength of the slice- selection gradient (Figure  4 a).

**Figure 5:**
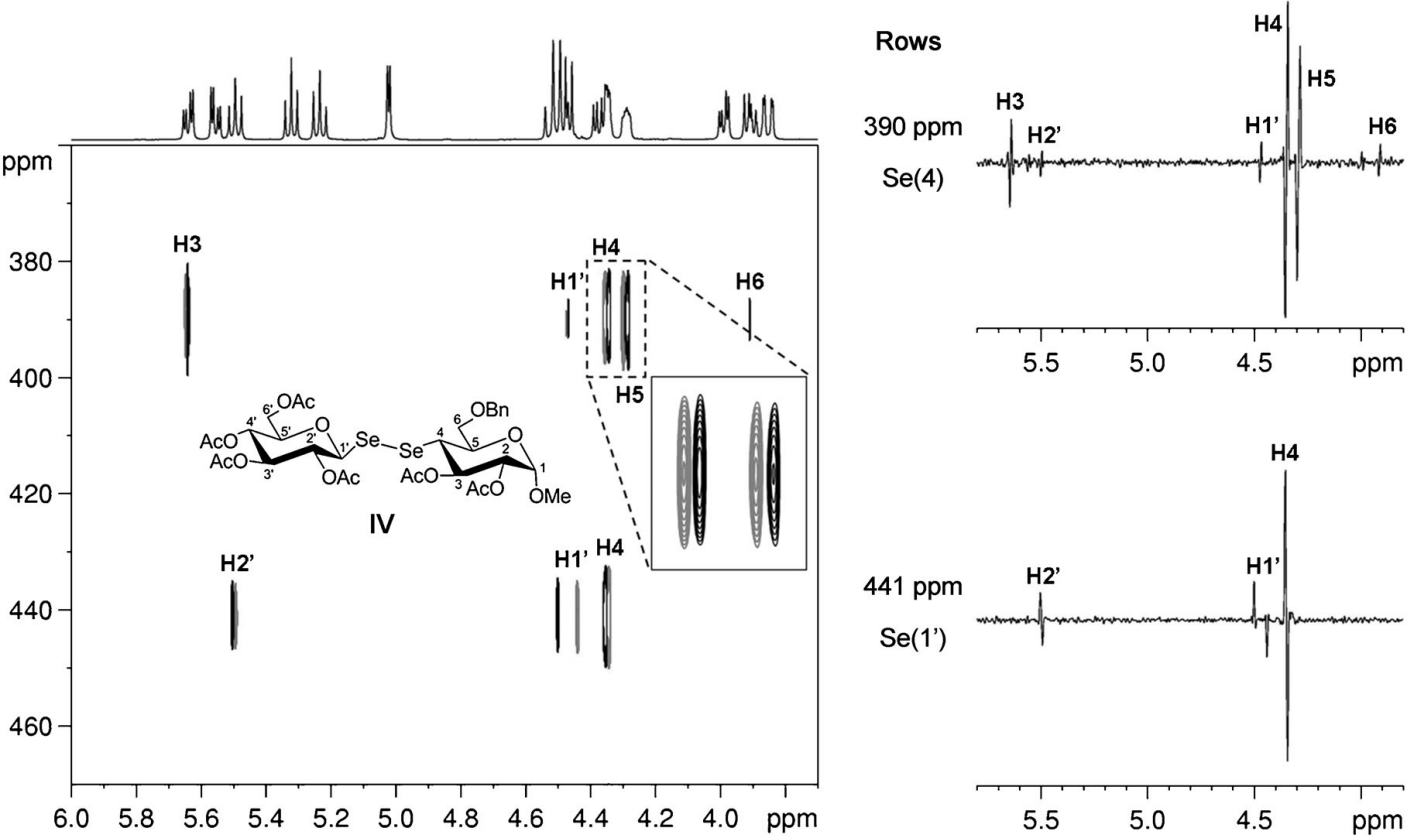
Representative broadband proton–proton-decoupled 2D CPMG-HSQMBC spectrum of diglycosyl diselenide IV. The extracted selenium traces shown next to the 2D spectrum nicely illustrate that the proposed method results in clean, pure absorptive antiphase doublets with splittings due solely to the desired multiple-bond heteronuclear couplings. The normal ^1^H spectrum can be seen above the 2D contour plot. The broadband proton–proton-decoupled CPMG-HSQMBC spectrum was recorded by using an RSNOB selective 180° proton pulse[47] of duration 46.64 ms and bandwidth 50 Hz under a slice-selection gradient (G6) of 0.5 % of the maximum gradient strength. The spectrum was acquired at 308 K in an experiment time of 18.3 h with spectral width in the ^1^H (^77^Se) dimension=9.9774 (140.0) ppm, number of *t*_1_ increments=32, number of *t*_2_ increments (i.e., number of FID chunks)=16, duration of FID chunk=16.56 ms, number of complex data points of constructed FID in ^1^H dimension=1600, relaxation delay=1.7 s, number of scans=48, and duration of long-range heteronuclear coupling evolution=81.7 ms.

The pseudo-2D version of the broadband proton–proton-decoupled CPMG-HSQMBC experiment was tested on a diglycosyl diselenide **IV** featuring an Se–Se bond in the interglycosidic bridge (Figure  5). The 1D traces extracted at the corresponding Se chemical shifts in Figure  5 illustrate that the proposed 2D experiment results in clean, purely absorptive antiphase doublets with splittings arising solely from multiple-bond heteronuclear couplings, and allows direct and precise measurement of ^*n*^*J*(^1^H,^77^Se) for molecules with more than one Se site.

**Figure 6:**
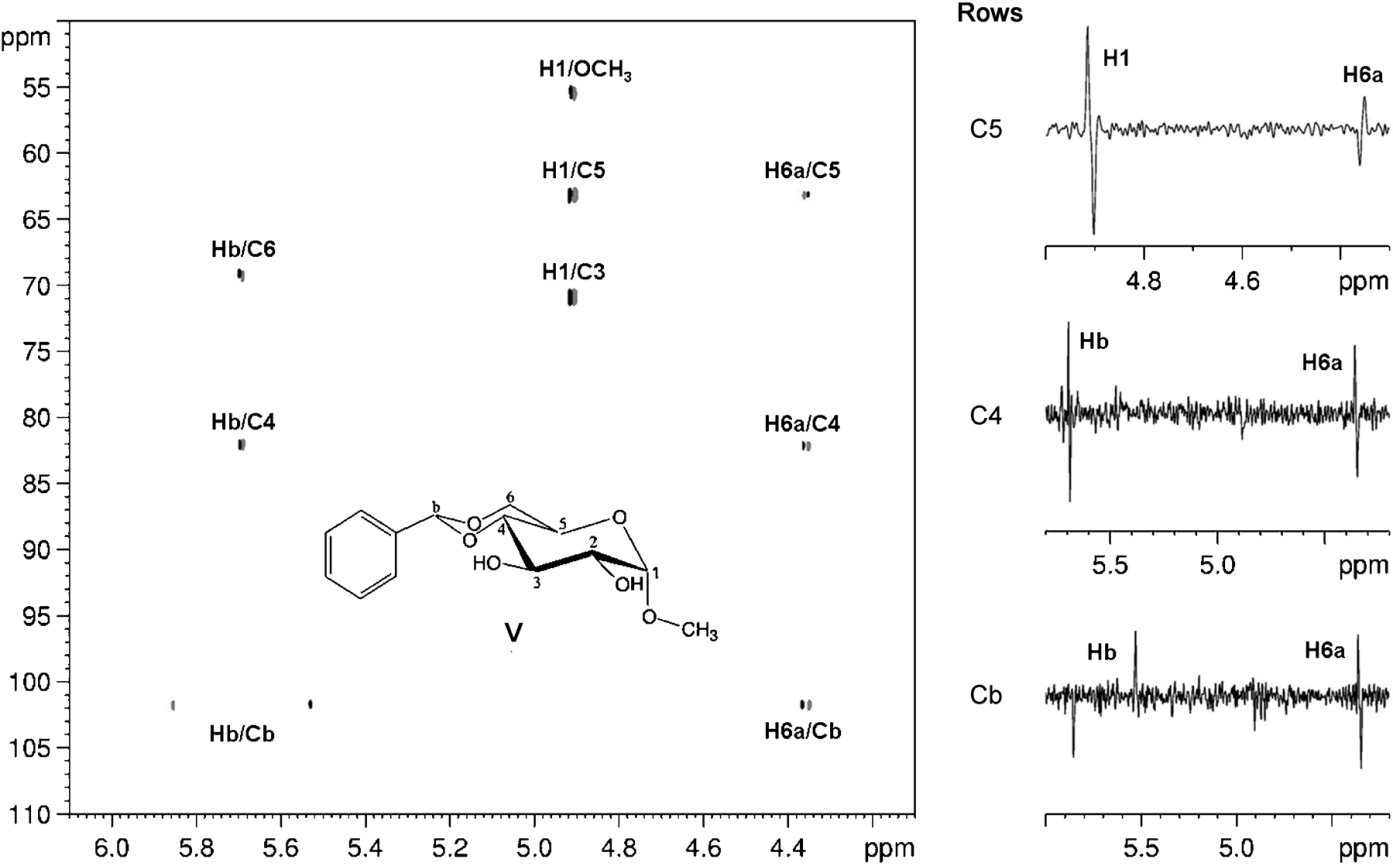
Partial contour plot of the broadband proton–proton-decoupled 2D CPMG-HSQMBC spectrum of V. The extracted carbon traces shown next to the 2D spectrum show pure absorptive antiphase doublets with splittings due solely to the heteronuclear couplings. The spectrum was recorded with an RSNOB selective 180° proton pulse[47] of duration 46.64 ms and bandwidth 50 Hz under a slice-selection gradient (G6) of 1 % of the maximum gradient strength. The spectrum was acquired in an experiment time of 38.7 h with spectral width in the ^1^H (^13^C) dimension=6.0370 (80.0) ppm, number of *t*_1_ increments=200, number of *t*_2_ increments (i.e., number of FID chunks)=16, duration of FID chunk=21.12 ms, number of complex data points of constructed FID in ^1^H dimension=2048, relaxation delay=1.7 s, number of scans=16, and duration of long-range heteronuclear coupling evolution=74.4 ms.

Finally, we demonstrate the utility of our method for the measurement of long-range ^1^H–^13^C coupling constants in the simple monosaccharide derivative **V** (Figure  6). Because of the significant sensitivity drop caused by the slice-selective proton pulse and the unfavorable abundance of the ^13^C nucleus, this experiment works only with highly concentrated (molar range) samples. However, recently the sensitivity of ZS-type experiments has been significantly improved. For example, by using multiple-frequency shaped pulses,[51] changing the offset of the selective shaped pulse after each scan,[52] and/or using advanced cryoprobes, the sensitivity of the broadband proton–proton-decoupled CPMG-HSQMBC experiment can be considerably enhanced. With these advances, the proposed method should become suitable for the determination of ^*n*^*J*(^1^H,^13^C) values under more realistic sample conditions.

## Conclusions

A ZS-based broadband proton–proton-decoupled CPMG-HSQMBC method has been devised for the precise and direct measurement of multiple-bond heteronuclear coupling constants. In the proposed experiment the undesired proton–proton splittings are removed from the heteronuclear multiplets, and thus the long-range heteronuclear couplings of interest can be determined from the resulting spectra simply by measuring the frequency differences between the peak maxima of pure antiphase doublets. However, when the coupling constant of interest is comparable to the proton line width, direct analysis of the antiphase signal can lead to overestimation of the magnitude of coupling. In such cases, separate recording of complementary in-phase data with a modified decoupled CPMG-HSQMBC sequence, including an additional refocusing period, allows the α/β multiplet components to be edited according to the well-known in-phase/antiphase (IPAP) approach.[53] The potential of the method has been demonstrated on molecules containing extended networks of mutually coupled protons. In such cases, additional multiplet fitting procedures would normally be required to extract long-range heteronuclear couplings from the complex signal patterns obtained in standard HSQMBC experiments. Our method also allows the measurement of a wide range of multiple-bond heteronuclear coupling constants in a single experiment. By using multiple-frequency shaped pulses and/or sensitive cryoprobes, the determination of heteronuclear long-range couplings for low-abundance nuclei may become feasible even for samples of modest concentration. Further improvement in sensitivity can be expected from incorporation of the recently developed PSYCHE[42] pulse sequence element or the instant (real-time) homonuclear broadband decoupling[30] methodology. Studies on implementing these approaches in the CPMG-HSQMBC sequence are under way.

## Experimental Section

All experiments were performed on a Bruker Avance II 500 spectrometer (Bruker BioSpin GmbH, Rheinstetten, Germany) equipped with a BBI or a TXI *z*-gradient probe. All spectra were processed with TopSpin 2.1, 2.5, or 3.0 (Bruker Biospin GmbH, Karlsruhe, Germany). The broadband proton–proton-decoupled pseudo-1D CPMG-HSQMBC method was tested on samples of 100 mg of **I** dissolved in 500 μL of CDCl_3_, 20.1 mg of **II** dissolved in 500 μL D_2_O, and 30 mg of **III** dissolved in 500 μL of CDCl_3_. The broadband proton–proton-decoupled pseudo-2D CPMG-HSQMBC spectra were acquired on samples of 117.5 mg of **IV** dissolved in 550 μL of C_6_D_6_ and 320 mg of **V** dissolved in 700 μL of [D_6_]DMSO. For all measurements the nominal temperature was set to 298 K, unless indicated otherwise.

To provide simultaneous composite π pulses on the ^1^H and X channels, power levels were carefully calibrated to give equal durations for proton and heteronucleus pulses. Spectra of selenium-containing compounds (Figures  2 and 5) were recorded with proton and selenium 90° pulses of 15 μs. Spectra of phosphorus-containing compounds (Figures  3 and 4) were collected with proton and phosphorus 90° pulses of 16 μs. The broadband proton–proton-decoupled ^1^H–^13^C CPMG-HSQMBC spectrum (Figure  6) was acquired with proton and carbon 90° pulses of 16 μs. However, when temperature-sensitive nuclei are studied and/or a cryoprobe is used, a CPMG cycle at reduced power level[18] (to give a 90° pulse of ca. 30 μs) is recommended to minimize heating of the sample and/or to protect probe electronics. Also, if compatible with the proton spectral parameters, the interpulse delays within the CPMG block can be increased (up to ca. 200–250 μs) for the same purpose.

The 2D and 3D raw data sets were processed with the Bruker AU program pshift (available at http://nmr.chemistry.manchester.ac.uk) to reconstruct the 1D and 2D interferograms. The pseudo-1D data were multiplied with a shifted sine-squared function, zero-filled to 16k, and then Fourier transformed to yield a spectral resolution of 0.1–0.3 Hz per point in the ^1^H dimension. Prior to 2D Fourier transformation the pseudo-2D data were multiplied with a shifted sine-squared function, zero-filled to 8k in the ^1^H dimension, and multiplied with a shifted sine-squared function, and zero-filled to 256 (Figure  5) and 512 (Figure  6) in the X dimension, before transformation to yield a spectral resolution of 0.2–0.4 Hz per point in the ^1^H dimension.

Bruker pulse sequence code is included in the Supporting Information. Other experimental details are given in the figure legends.

## Supporting Information

As a service to our authors and readers, this journal provides supporting information supplied by the authors. Such materials are peer reviewed and may be re-organized for online delivery, but are not copy-edited or typeset. Technical support issues arising from supporting information (other than missing files) should be addressed to the authors.

miscellaneous_information
