# Supplementary material for: Precise Measurement of Long-Range Heteronuclear Coupling Constants by a Novel Broadband Proton–Proton-Decoupled CPMG-HSQMBC Method
Source: Chemistry. 2015 Jan 8;21(8):3472–9. doi: 10.1002/chem.201405535 (PMC4338765; doi:10.1002/chem.201405535)
Supplement: Supplementary file 1 — miscellaneous_information [file chem0021-3472-SD1.pdf]

CHEMISTRY
A European Journal

Supporting Information

© Copyright Wiley-VCH Verlag GmbH & Co. KGaA, 69451 Weinheim, 2015

Precise Measurement of Long-Range Heteronuclear Coupling Constants by a Novel Broadband Proton–Proton-Decoupled CPMG-HSQMBC Method

István Timári,[a] Tünde Z. Illyés,[b] Ralph W. Adams,[c] Mathias Nilsson,[c] László Szilágyi,[b] Gareth A. Morris,[c] and Katalin E. Kövér*[a]

chem_201405535_sm_miscellaneous_information.pdf

Bruker pulse sequence code:

```
;ek_ti_cpmg_hsqmbc2D_ps
;
;broadband proton-proton - decoupled CPMG-HSQMBC
;pseudo 2D experiment used for precise and accurate determination of long-range heteronuclear
;coupling constants
;phase sensitive using Echo/Antiecho gradient selection
;using CPMG for polarization transfer to avoid evolution of J(HH)
;using composite X and 1H 180 pulses in CPMG
;using Zangger-Sterk based broadband proton-proton decoupling scheme
;J(HH) is refocussed at the centre of chunk
;pseudo 2D data can be reconstructed using the 'pshift' macro available at
;http://nmr.chemistry.manchester.ac.uk
;
;This pulse sequence is part of:
;I. Timari, T.Z. Illyes, R.W. Adams, M. Nilsson, L. Szilagyi, G.A. Morris, K.E. Kover:
;"Precise measurement of long-range heteronuclear coupling constants by a novel broadband
;proton-proton - decoupled CPMG-HSQMBC method"
;Chem. Eur. J. 2014
;
;Further relevant publications:
;K.E. Kover, G. Batta, K. Feher, J. Magn. Reson. 2006, 181, 89-97.
;J.A. Aguilar, S. Faulkner, M. Nilsson, G.A. Morris, Angew. Chem. Int. Ed. 2010, 49, 3901-
;3903.
;
;Bruker Avance II version, July 31, 2014

;$CLASS=HighRes
;$DIM=2D
;$TYPE=
;$SUBTYPE=
;$COMMENT=


#include <Avance.incl>
#include <Grad.incl>
#include <Delay.incl>

define delay tauA
define delay tauB
define delay tauC


"in10=inf1/2"
"in0=inf2/2"

"d10=0"
"d0=3u"

"p2=p1*2"

"tauA=in10/2-p16-d16-50u"
"tauB=in10-p16-d16-300u"
"tauC=in10/2-p16-d16-350u-(dw*2)-(dw*2*cnst4)-de"

"p4=p3*2"
"d11=30m"
"d13=3u"
"d7=d13+p16+d16+4u"
"d20=p16+d16+p2+d0*2"

aqseq 312


1 ze
2 d1 do:f2
  d11 pl1:f1
3 p1 ph1


4  d15 pl2:f2                  ;CPMG-sequence for polarization transfer with XY-16 phase cycle
  (p1 ph20) (p3 ph20):f2
   3u
```

```
  (p2 ph21) (p4 ph21^):f2      ;^ increment phase pointer of ph21
   3u
  (p1 ph20) (p3 ph20^):f2      ;^ increment phase pointer of ph20 - composite 1H, X pulse
   d15                         ;d15=120-150us
   lo to 4 times l1            ;p1 should be calibrated to p1=p3 at pl1!!!
                               ;l1 = multiple of 16!

                               ;long-range coupling evolution = (2*d15+2*p4+6)*l1 (ca.50-90ms)

  (p1 ph2)
  d13 UNBLKGRAD
  p16:gp3                   ;gpz3=19 purging
  d16
  (p3 ph3):f2
  d0
  p2 ph5
  d0
  p16:gp1                   ;gpz1=80 for 13C, coherence selection
  d16

  (p3 ph14):f2              ;comp. X 180 pulse
  (p4 ph4):f2
  (p3 ph14):f2

  d20

 (p3 ph4):f2
  d13
  p16:gp4                   ;gpz4=10 purging
  d16
  (p1 ph16)
  d7
  (p2 ph19)
  d13
  p16:gp2*EA               ;gpz2=20.1 for 13C, echo-antiecho coherence selection
  d16
  4u


  d10                     ; Incremented delay for broadband proton-proton decoupling protocol

  tauA
  50u
  p16:gp5*1.0             ; CTP, +1.0*
  d16

  p2 ph17                 ; 180 (H)

  tauB
  p16:gp5*4.0             ; CTP, +4.0*
  d16

  300u gron6              ; Slice selection gradient On
  p12:sp2:f1 ph18:r       ; 180 (selective)
  100u groff              ; Slice selection gradient Off
  200u

  p16:gp5*3.0             ; CTP, +3.0*
  d16
  50u BLKGRAD
  tauC
  d10                     ; incremented delay

5 go=2 ph31
  30u
  d1 do:f2 mc #0 to 2
  F1QF(id10)
  F2EA(rd10 & igrad EA, id0)

6 exit

ph1=0
ph2=1
ph11=2
ph12=3
ph20=1 2 1 2 2 1 2 1 3 0 3 0 0 3 0 3
ph21=0 1 0 1 1 0 1 0 2 3 2 3 3 2 3 2
```

```
ph3=0 2
ph4=0 0 2 2
ph14=1 1 3 3
ph5=0

ph16=0
ph19=1

ph17=0

ph18=0 0 0 0 1 1 1 1

ph31=0 2 2 0 2 0 0 2


;pl1 : f1 channel - power level for pulse (default)
;pl2 : f2 channel - power level for pulse (default)
;sp2: selective pulse power level
;spoffs2: selective pulse offset (0 Hz)
;spnam2:  file name for selective pulse
;p1 : f1 channel -  90 degree high power pulse
;p2 : f1 channel - 180 degree high power pulse
;p3 : f2 channel -  90 degree high power pulse
;p4 : f2 channel - 180 degree high power pulse
;p12: selective 180 pulse width
;p16: homospoil/gradient pulse
;d0 : incremented delay                 [3 usec]
;d1 : relaxation delay; 1-5 * T1
;d7: =d13+p16+d16+4u
;d10: incremented delay, set initial value to 0 s
;d11: delay for disk I/O                             [30 msec]
;d13: short delay                                    [3 usec]
;d15: interpulse delay
;d16: delay for homospoil/gradient recovery
;d20: =p16+d16+p2+d0*2
;l1: loop for CPMG
;cnst4: number of points to drop when collecting FID
;NS: number of scans
;DS: number of dummy scans, >= 32
;td1: number of chunks to acquire
;FnMODE1: QF
;FnMODE2: Echo-Antiecho
;gpz1: 80%
;gpz2: 20.1% for C-13, 8.1% for N-15
;gpz3: purging gradient (19%)
;gpz4: purging gradient (10%)
;gpz5: CTP gradient (18%)
;gpz6: slice selection gradient
;
;use gradient files:
;gpnam1: SINE.100
;gpnam2: SINE.100
;gpnam3: SINE.100
;gpnam4: SINE.100
;gpnam5: SINE.100
```
